# Prostate cancer recurrence in vas deferens – fusion image guide as an important tool in dignosis

**DOI:** 10.1590/S1677-5538.IBJU.2017.0071

**Published:** 2018

**Authors:** Leonardo Guedes Moreira Valle, Antônio Rahal, Priscila Mina Falsarella, Juliano Ribeiro de Andrade, Oren Smaletz, Akemi Osawa, Rodrigo Gobbo Garcia

**Affiliations:** 1 Departamento de Radiologia Intervencionista, Hospital Israelita Albert Einstein, São Paulo, Brasil;; 2 Departamento de Oncologia, Hospital Israelita Albert Einstein, São Paulo, Brasil;; 3 Departamento de Medicina Nuclear e Radiologia, Hospital Israelita Albert Einstein, São Paulo, Brasil

**Keywords:** Prostatic Neoplasms, Vas Deferens, Therapeutics

## Abstract

The biochemical recurrence after local treatment for prostate cancer is an often challenging condition of clinical management. The aim of this report is to demonstrate the importance of the association of various imaging methods in the identification and subsequent accurate percutaneous biopsy in patients with recurrence of prostate cancer, especially in unusual sites.

An 86 years old male with biochemical recurrence, during radiological investigation a PET-MRI was noted the presence of an asymmetry of the vas deferens with PSMA-^68^Ga uptaken, suggesting the recurrence. A percutaneous fusion biopsy with PET-MRI and ultrasound was performed using transrectal access using ultrasound confirming infiltrating adenocarcinoma of the wall of the vas deferens, compatible with neoplastic prostate recurrence.

The fusion image technique combines the real–time view of the US to the possibility of higher definition and higher specificity, methods more anatomical detail as tomography and magnetic resonance imaging, simultaneously.

High resolution acquired in PET / MR associated with image fusion allows orientation procedures, even in areas of difficult access, with greater accuracy than conventional techniques.

## INTRODUCTION

Up to 35% of patients with localized treatment for prostate cancer (radical prostatectomy or radiation therapy) ultimately present with a biochemical recurrence, which means a prostate specific antigen (PSA) elevation without abnormal conventional imaging studies. The biochemical recurrence after local treatment is an often challenging condition of clinical management (1).

An emerging tool for the topographic diagnosis of lesions in patients with biochemical recurrence is the use of prostate-specific membrane antigen (PSMA) PET scan associated with the magnetic resonance imaging (MRI), promoting high tumor contrast associated with high resolution image (2), identifying possible focus of tumor recurrence. This association of images featuring the PET-MRI exam differs in order to provide diagnostic accuracy based on aspects of both functional and anatomical.

The aim of this report is to demonstrate the importance of the association of various imaging methods in the identification and subsequent accurate percutaneous biopsy in patients with recurrence of prostate cancer, especially in unusual sites. Similarly, we aim to report an uncommon presentation of recurrence of prostate cancer in the vas deferens, location which particularly benefited from the above-mentioned imaging methods combination.

## CASE REPORT

A 86 years-old male has been followed for elevated PSA levels after his prostatectomy fifteen years ago. He has already undergone salvage external beam radiation therapy with no success and has been on different hormonal manipulations since then. His current PSA was 21 with a doubling time of 1 month with a testosterone level of 23ng/dl.

**Figure 1:**
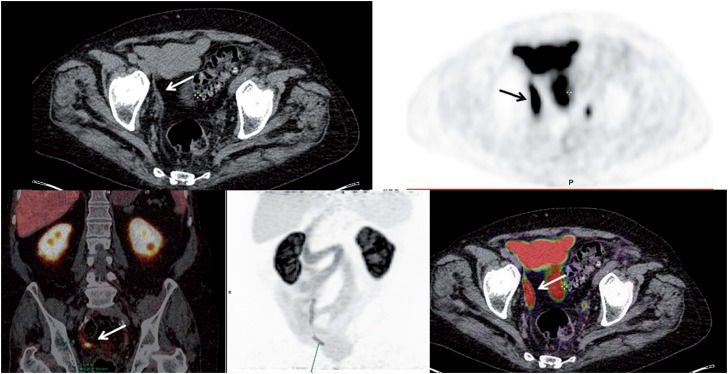
Anomalous accumulation of PSMA-68Ga in the right vas deferens. That has increased dimension compared to the contralateral, suggestive of secondary neoplastic lesion found, considering the underlying disease.

Abdominal/pelvic tomography observed nonspecific thickening of the right vas deferens. In additional radiological investigation PET-MRI noted the presence of an asymmetry of the vas deferens with PSMA-^68^Ga uptake, suggesting recurrence (Figure-1). Given the rarity of this presentation, it was required histologic sampling for appropriate treatment planning.

**Figure 2:**
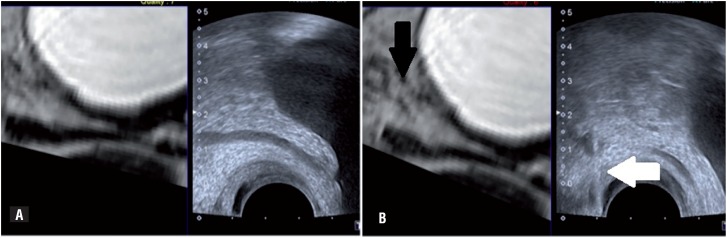
A) Tomography and ultrasound images fusion, showing images synchronization. B) On the left, arrow (black) shows the position of the thickened vas deferens (CT); on the right, the biopy needle into the target lesion (white arrow).

**Figure 3:**
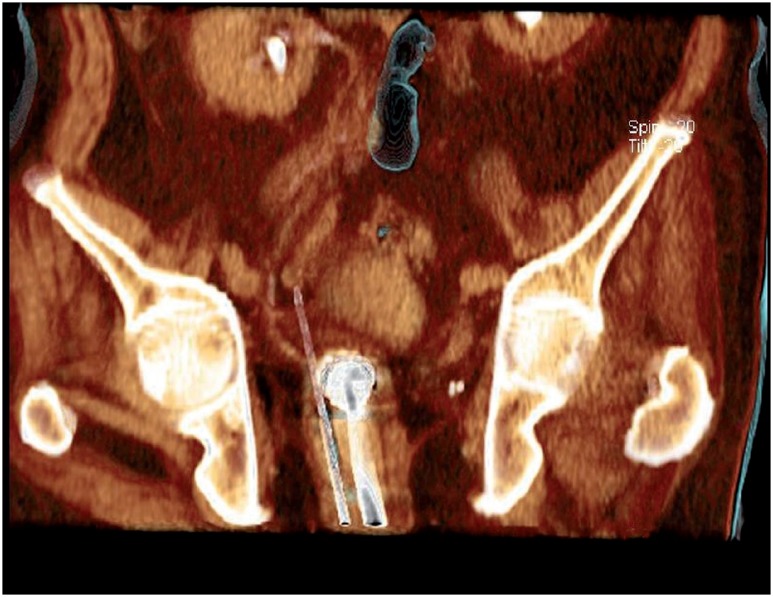
Tomographic reconstruction positioning of the transrectal probe and biopsy needle to the target lesion.

A percutaneous fusion biopsy with PET-MRI and ultrasound was performed using transrectal access using ultrasound (Toshiba Aplio 500 Platinum-Toshiba American Medical Systems, Tustin, CA) and probe with guide prostate biopsies. An 18G automatic needle (Acecut-TSK Laboratory-Japan) was positioned into the lesion guided by real time fusion images and confirmed the position by tomography (Somatom Definition AS 40-slice, Siemens, Berlin, Germany) (Figure-2 and Figure-3).

Five samples were collected, with immediate analysis by the pathologist, confirming the presence of atypical cells.

Control tomography and transrectal ultrasound showed no immediate complications and the patient was discharged after 4 hours of observation and micturition without hematuria.

Final pathological analysis of the sample showed infiltrating adenocarcinoma of the wall of the vas deferens and the adjacent adipose tissue compatible with neoplastic prostate recurrence. Due to prior external beam radiation therapy, he was started on Enzalutamide in addition to androgen deprivation associated with radiation therapy directed at the focus of tumor recurrence.

Institutional review board approval for case report is not required in our institution.

## DISCUSSION

The biochemical recurrence is challenging, the rising of PSA level is not necessary associated with imminent death from disease, but patients concern about this rising and eager may hinder treatment. Precipitated interpretations may cause prematurely administration of androgen deprivation therapy. It is known that androgen blockage is associated with side effects such as impotence, bone loss, metabolic syndrome and increased risk of cardiovascular disease; it is worsened if the evolution of the disease is not associated with changes in survival (3).

Hence, there is the quest for new diagnostic methods with high specificity and sensitivity to locate and predict the extent of the disease in case of biochemical recurrence. Other methods associated with membrane receptors have already been tried, but did not achieve the expected result.

Positron emission tomography-magnetic resonance imaging (PET-MRI) is an image of a hybrid technique that combines morphological and functional information with high accuracy in diagnosis of primary and metastatic tumors. The prostate specific membrane antigen (PSMA) is a transmembrane protein with increased expression in neoplastic prostate cells compared to benign glandular cells. Currently, some studies (4) have demonstrated an increase in efficiency in the early anatomical diagnosis in patients with biochemical recurrence of prostate cancer in the PET with PSMA compared with other radiotracers.

The fusion image technique combines the real-time view of the US to methods of higher definition and specificity with better anatomic detail. In addition, the detection of clinically significant prostate cancer in MRI helps to detect the same disease in biopsy results (5).

Although Lee et al. did not find significant difference between the use of fusion biopsy and systematic biopsy in detection rate of any-grade prostate cancer (6), Mariotti et al. had a consistently result in detecting more intermediate-to-high risk using fusion biopy (7).

Our experience is in accordance with the literature, where it is observed a 13.5% upgrade for MRI-US fusion biopsy over systematic biopsy (8), that shows the importance of fusion biopsy.

This case demonstrates the effectiveness of PET-MRI/PSMA in identifying the location of probable recurrence and, in combination with fusion biopsy, allowed the accurate diagnosis of the site of recurrence, contributing to the better treatment of the condition.

## CONCLUSIONS

High resolution acquired in PET/MR associated with image fusion allows orientation procedures, even in areas of difficult access, with greater accuracy than conventional techniques.
